# Retinal pigment epithelial cell multinucleation in the aging eye – a mechanism to repair damage and maintain homoeostasis

**DOI:** 10.1111/acel.12447

**Published:** 2016-02-15

**Authors:** Mei Chen, Dinusha Rajapakse, Monika Fraczek, Chang Luo, John V. Forrester, Heping Xu

**Affiliations:** ^1^ Centre for Experimental MedicineSchool of Medicine, Dentistry & Biomedical SciencesQueen's University Belfast97 Lisburn RoadBelfastBT9 7 BLUK; ^2^ Section of Immunology and InfectionDivision of Applied MedicineSchool of Medicine and DentistryInstitute of Medical ScienceUniversity of AberdeenForesterhillAberdeenAB25 2ZDUK; ^3^ Ocular Immunology ProgramCentre for Ophthalmology and Visual ScienceThe University of Western AustraliaPerthWA6009Australia; ^4^ Centre for Experimental ImmunologyLions Eye InstituteNedlandsWA6009Australia

**Keywords:** aging, cytokinesis, multinucleation, phagocytosis, photoreceptor outer segments, retinal pigment epithelium

## Abstract

Retinal pigment epithelial (RPE) cells are central to retinal health and homoeostasis. Dysfunction or death of RPE cells underlies many age‐related retinal degenerative disorders particularly age‐related macular degeneration. During aging RPE cells decline in number, suggesting an age‐dependent cell loss. RPE cells are considered to be postmitotic, and how they repair damage during aging remains poorly defined. We show that RPE cells increase in size and become multinucleate during aging in C57BL/6J mice. Multinucleation appeared not to be due to cell fusion, but to incomplete cell division, that is failure of cytokinesis. Interestingly, the phagocytic activity of multinucleate RPE cells was not different from that of mononuclear RPE cells. Furthermore, exposure of RPE cells *in vitro* to photoreceptor outer segment (POS), particularly oxidized POS, dose‐dependently promoted multinucleation and suppressed cell proliferation. Both failure of cytokinesis and suppression of proliferation required contact with POS. Exposure to POS also induced reactive oxygen species and DNA oxidation in RPE cells. We propose that RPE cells have the potential to proliferate *in vivo* and to repair defects in the monolayer. We further propose that the conventionally accepted ‘postmitotic’ status of RPE cells is due to a modified form of contact inhibition mediated by POS and that RPE cells are released from this state when contact with POS is lost. This is seen in long‐standing rhegmatogenous retinal detachment as overtly proliferating RPE cells (proliferative vitreoretinopathy) and more subtly as multinucleation during normal aging. Age‐related oxidative stress may promote failure of cytokinesis and multinucleation in RPE cells.

## Introduction

The retinal pigment epithelium is a monolayer of cells located between Bruch's membrane and the photoreceptor outer segments (POS) of the retina. RPE cells are critical for retinal homoeostasis and essential for the visual cycle (Strauss, 2005). Critical functions include outer blood–retinal barrier (oBRB) properties; transport of oxygen and nutrients from the choroid to the outer layers of the retina and removal of metabolic waste from the outer retina to choroid [reviewed in (Strauss, 2005)]; and maintenance of an immune‐regulatory microenvironment within the subretinal space (Nussenblatt *et al*., 2014; Ozaki *et al*., 2014). Some aspects of RPE cell biology have been reviewed recently (Pfeffer & Philp, 2014).

The RPE is considered a terminally differentiated, ‘postmitotic’ cell. During normal aging, RPE cell numbers decline (Gao & Hollyfield, 1992) but, in health, the monolayer is maintained, suggesting the existence of a repair or compensatory mechanism. Previous studies have noted a variable increase in RPE cell size as well as multinucleation with age (Ts'o & Friedman, 1967, 1968), but the significance of these observations is unclear. Giant cell formation with multinucleation is a normal feature of some cells such as osteoclasts and syncytiotrophoblasts (Park & Askin, 2013; Oh *et al*., 2014) and also occurs during pathological processes such as foreign body giant cell formation in macrophages (Vignery, 2005; MacLauchlan *et al*., 2009), and in microglial cells in some forms of neurodegeneration (Lee *et al*., 1993; Hornik *et al*., 2014). Giant cell formation and multinucleation, at least in osteoclasts, are formed by cell fusion, but other mechanisms may also explain cells with multiple nuclei such as phagocytosis of live cells or failure of cytokinesis in dividing cells (Hornik *et al*., 2014).

Multinuclear cell formation in RPE cells might therefore represent a pathological condition in a cell under stress or a healthy cell which has adapted to its changing environment for instance with age. Therefore, it is important to determine whether such changes indicate continued health of the RPE cell or predicate impending RPE cell damage and death as the latter are the hallmark of age‐related macular degeneration (AMD), one of the most common retinal degenerations causing blindness (Lim *et al*., 2012). The aim of this study was to quantify the cell biological changes in the aging mouse RPE and to correlate these changes with possible functional alterations. Our data show that with age, RPE cells increased in size and became multinucleated. Multinuclear RPE cells were functionally active and retained their phagocytic capability. Importantly, we show that phagocytosis of POS by RPE cells suppressed RPE cell proliferation *in vitro* while promoting multinucleation, indicating a central function for POS in regulating RPE cell behaviour. Moreover, the mechanism whereby POS induced RPE multinucleation appeared to be through disruption of cytokinesis without altering RPE functionality.

## Results

### The decline in RPE cell number is greater than the reduction in RPE cell nuclei with age

**Figure 1:**
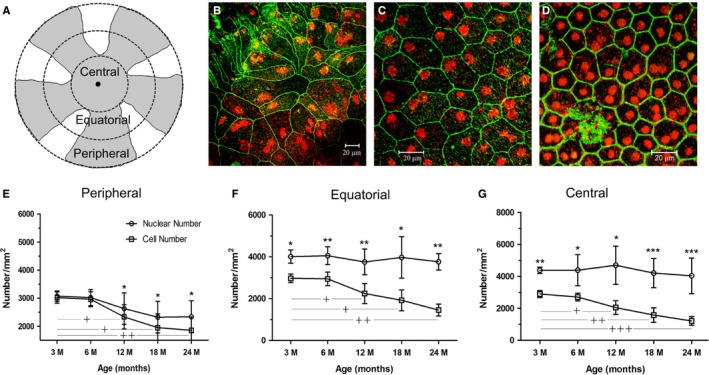
RPE cells in mice of different ages. RPE/choroid/sclera flat mounts were stained with phalloidin (for F‐actin, green) and PI (red) and imaged by confocal microscopy. (A) a schematic graph showing different geographic locations of RPE flat mounts used in image analysis. (B–D) typical confocal images of RPE flat mounts from a 6‐month‐old mouse showing RPE cells in the peripheral (B), equatorial (C) and central (D) regions. (E–G) the number of RPE cells and the number of RPE nuclei in different regions of the eye from different ages of mice. *, *P *< 0.05; **, *P *< 0.01, ***, *P *< 0.001 compared to cell number at the same age time point. †, *P *< 0.05; ††, *P *< 0.01 ††††, *P *< 0.001 compared to the cell number of the 3 m age group. *N *≥ 8.

Using the optic disc as a reference point, we divided RPE flat mounts equally into three regions: the peripheral region, the equatorial region and the central region (Fig. 1A). RPE cells in the peripheral region (Fig. 1B) vary in size and shape. Some cells are elongated, and others have irregular or cobblestone‐like shapes (Fig. 1B). The RPE cells in the equatorial and central regions are more uniform with a pentagonal or hexagonal shape (Fig. 1C,D). An age‐dependent reduction in RPE cell numbers was observed in all regions (Fig. 1E–G). Interestingly, we observed many binucleate and multinucleate RPE cells (Fig. 1E–D), particularly in mice older than 6 months (Fig. 1B–D). Moreover, the number of nuclei was significantly greater than the number of cells at all ages of mice in the equatorial and central regions (Fig. 1E–G). However, an age‐related reduction in the number of nuclei was only observed in the peripheral region (Fig. 1E).

### Binucleate and multinucleate RPE cells in mice of different ages

Regional differences in the proportions of single nucleus vs. multinucleate RPE cells were quite marked. At all ages, the peripheral retina contained the highest percentage of mononucleate and the lowest percentage of multinucleate cells (1.7–20.5%, Fig. S1A–B). The highest percentage of multinucleate cells occurred in the central retina at all ages (33.6–79.7%, Fig. S1B,F). Remarkably, the percentage of multinucleate cells in 24‐month‐old mice reached levels of nearly 80% in the central retina while remaining at levels of around 20% in the peripheral retina (Fig. S1B). Intermediate levels of multinucleate RPE cell were observed in the equatorial retina (Fig. S1B,E).

The majority of multinucleate RPE cells in all regions contained two nuclei while cells with multiple nuclei (>3, Fig. SF) were less frequently observed. However, significantly greater numbers of cells with ≥3 nuclei were observed in mice aged between 12 and 24 months and were predominately located in the central retina (*P *< 0.01 compared to peripheral, Fig. S1C). Such cells were also significantly larger than cells with single or two nuclei (Fig. S1G).

### Age‐related morphological change in RPE cells

In general, the size of peripheral RPE cells was larger than that of equatorial and central RPE cells (Figs S1D–F and S2A–B). There was also an age‐dependent increase in the overall cell size and in the distribution range of cell size at all regions (Fig. S2A–D). Occasionally, cells occupying areas as large as 2000–2300 μm^2^ were observed in aged mice (Fig. S2C,D).

**Figure 2:**
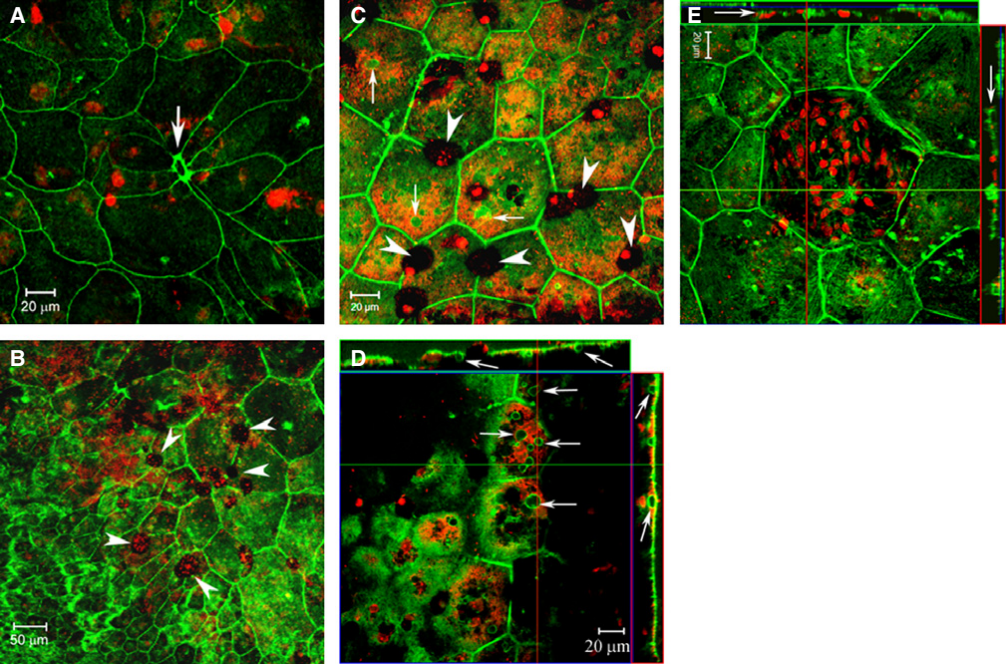
RPE cell morphological changes in aged mice. RPE/choroid/sclera flat mounts from 18‐month (A, B)‐ and 24‐month(C–D)‐old mice were stained with phalloidin (green) and PI (red) and imaged by confocal microscopy. (A) a F‐actin^hi^ lesion (arrow) is surrounded by 8 RPE cells, and a few of the cells have an appearance of cell body extension. This type of lesion was frequently observed in mice older than 6 months. (B) a confocal image from a 18‐month‐old mouse shown the transition between areas with normal RPE cells (lower left) and giant RPE cells (upper right). A few pigmented cells (arrowheads) were seen on RPE surface. (C–D) high‐magnification images showing giant RPE cells with diffused PI staining and multiple small F‐actin^+^ intracellular vacuoles (small arrows). The vacuoles appear to be connected with cytoplasmic membrane in z‐stack images (D). Many pigmented cells were observed on the RPE surface (arrowheads in C). E, a giant RPE cell with multiple nuclei in a 24‐month‐old mouse.

Certain patterns emerged in relation to the distribution of large cells. For instance, small collections of large RPE cells surrounding a discrete F‐actin^+^ round/oval‐shaped lesion were frequently observed in mice older than 6 months (Fig. 2A). In addition, in mice aged between 18 and 24 months, small pigmented cells (presumably damaged RPE cells or infiltrating macrophages containing phagocytosed melanin (Xu *et al*., 2008), Fig. 2B,C) were frequently observed located on the surface of RPE cells, in the areas where the RPE cells were very large and contained multiple intracellular vacuoles (Fig. 2C,D). These giant RPE cells stained diffusely with propidium iodide, a sign of cell death, and lacked evidence of definitive nuclei (Hesse *et al*., 2012) (Fig. 2C,D). An example of a giant RPE cell with multiple small condensed PI staining bodies in a 24‐month‐old mouse retina is shown (Fig. 2E).

### Alpha‐tubulin expression and BrdU uptake in mice of different ages

**Figure 3:**
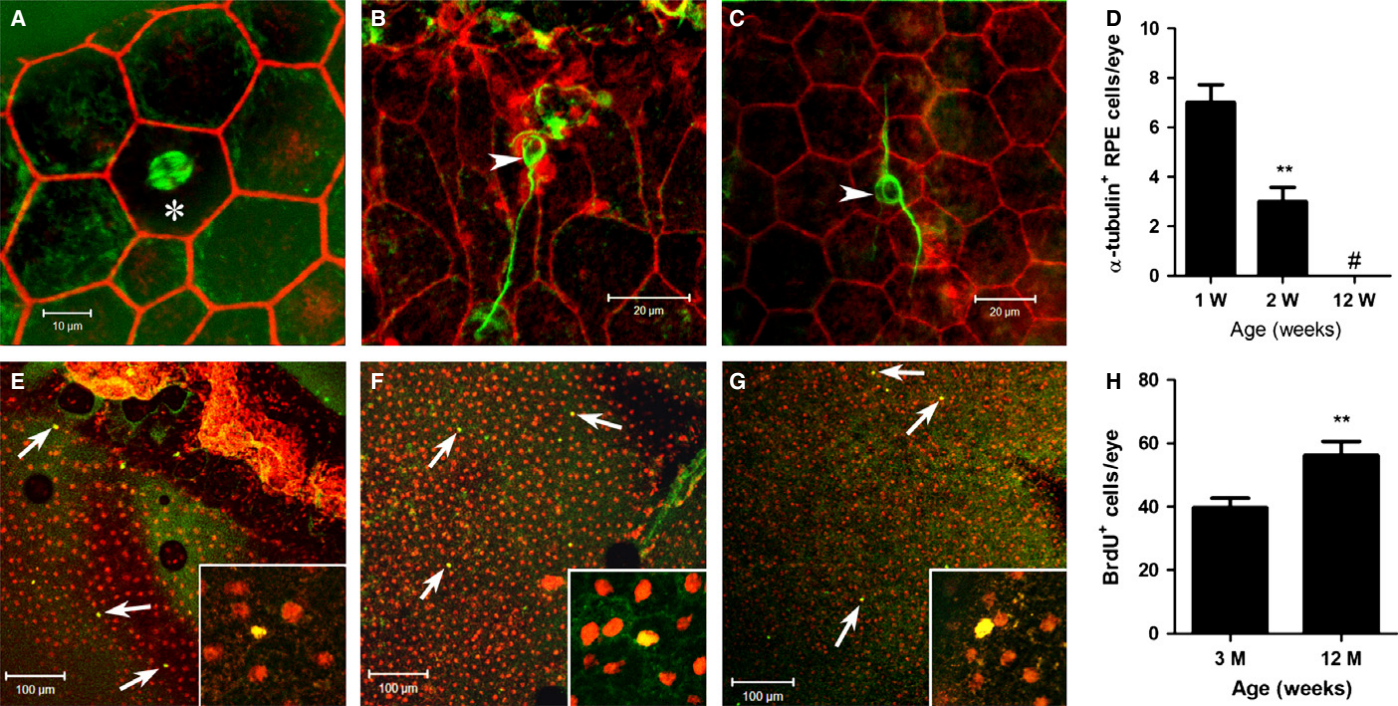
Detection of α‐tubulin^+^ RPE cells and BrdU^+^ RPE cells in mice of different ages. (A–D) RPE/choroid/sclera flat mounts from 1‐week(A)‐ and 12‐week‐old (B, C) mice were stained with phalloidin (red) and α‐tubulin (green) and imaged by confocal microscopy. An α‐tubulin‐expressing RPE cell is shown in A (asterisk), and α‐tubulin‐expressing tubular structures was detected in the peripheral (B) and central (C) RPE flat mounts in 12‐week‐old mice. (D) histogram shown the number of α‐tubulin expressing RPE cells at different ages. **, *P *< 0.01 compared to 1‐week‐old mice, #, value not detected. *N *= 6 mice. E‐H, 3‐ and 12‐month‐old mice were injected with BrdU for 7 days. RPE flat mounts were stained for BrdU (green) and PI (red) and examined by confocal microscopy. (E–G) confocal images from 12‐month‐old mice showing BrdU^+^ cells in the peripheral (E), equatorial (F) and central (G) regions. H, histogram showing the total number of BrdU^+^ cells in each eye. **, *P *< 0.01 compared to 3‐month‐old mice, unpaired Student's *t*‐test. *N *= 6 mice.

Alpha (α)‐ and β‐tubulins are essential for microtubule‐based spindle formation during mitosis. RPE flat mounts stained with antibodies to tubulin revealed α‐tubulin spindles inside RPE cells in mice under 2 weeks old (Fig. 3A). An average of 7 α‐tubulin^+^ cells (5–12 cells/retina, Fig. 3A) in 1‐week‐old mice and 3 (1–5 cell/eye) in 2‐week‐old mice, but none in mice older than 2 weeks were detected (Fig. 3B–D). Interestingly, α‐tubulin^+^ tubule‐like structures on the surface of some RPE cell were commonly detected in RPE flat mounts of adult mice in all three regions, that is peripheral (Fig. 3B), equatorial (Fig. 3C) and central (data not shown) regions. During the anaphase of mitosis, spindle fibres draw chromosomes to the opposite poles of the cell in the process of cytokinesis to form two daughter cells. Our data suggest that RPE proliferation (i.e. full cell division: DNA synthesis, mitosis and cytokinesis) occurs in mice during the first 2 weeks of life, but the full process is not evident after this time.

Evidence of DNA synthesis was also sought using BrdU labelling *in vivo*. When BrdU was injected into 3‐ and 12‐month‐old mice for a consecutive period of 7 days, a small number of BrdU^+^ cells (20–90 cell/eye) were detected in different regions of the RPE flat mounts monolayers although the peripheral RPE, particularly, around the ciliary body (Fig. 3E) contained more cells than the equatorial (Fig. 3F) and central (Fig. 3G) RPE. Interestingly, more BrdU^+^ cells were detected in 12‐month‐old mice compared to that in 3‐month‐old mice (Fig. 3H). Our results suggest that active DNA synthesis exists at low levels in RPE cells in adult mouse eyes, despite the lack of evidence for full cell division.

### Effect of photoreceptor out segments on RPE cell proliferation *in vitro*

**Figure 4:**
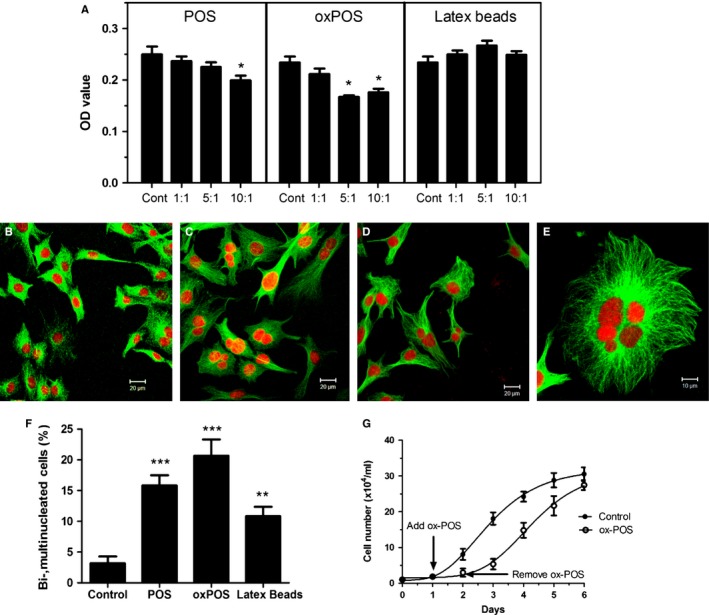
The effect of photoreceptor outer segment (POS) on RPE cell proliferation and multinucleation. B6‐RPE07 mouse RPE cells were treated with different concentrations of POS or oxidized POS (oxPOS) or latex beads for 48 h. (A) cell proliferation was detected by MTT assay. *, *P *< 0.05 compared to control group, One‐way ANOVA followed by Dunnett's multiple comparison test. *N *= 3. (B–F) following treatment, RPE cells were fixed with ethanol and stained for α‐tubulin and PI. The cells were imaged by confocal microscopy. (B) confocal image from control nontreated cells. (C) confocal image from oxPOS (5:1)‐treated cells. (D) confocal image from latex beads (5:1)‐treated cells. (E) a high‐magnification image shown a RPE cell with 6 nuclei following oxPOS treatment. (F) histogram shown the percentage of binucleated and multinucleated RPE cells following different treatments. **, *P *< 0.01, ***, *P *< 0.001 compared to controls. One‐way ANOVA followed by Dunnett's multiple comparison test. *N *= 3. (G) Growth curve of B6‐RPE07 mouse RPE cells with and without ox‐POS treatment. B6‐RPE07 cells were cultured in 24‐well plates at 1 × 10^4^ ml^‐1^. Twenty‐four hours later (day 1), one group of cells was treated with 5 × 10^4^ ml^−1^ ox‐POS for 24 h. At day 2, ox‐POS was removed from the culture. Cell numbers from each group were counted from three wells each day.

As indicated above, RPE cells *in vivo* are considered terminally differentiated (postmitotic) with little evidence of proliferation in adult eyes and our data support this view. However, RPE cells in pathological conditions such as long‐standing retinal detachment (PVR) actively proliferate and induce extensive periretinal scar tissues, a complication of long‐standing retinal detachment, and RPE cells *in vitro* show strong proliferative activity. We were interested to determine what role POS may play in the regulation of RPE cell proliferation and/or multinucleation. When mouse RPE cells (primary or B6‐RPE07) were exposed to POS or oxPOS for 48 h, a dose‐dependent suppression of cell proliferation was observed with oxPOS showing a stronger effect than POS (Fig. 4A). In contrast, exposure to latex beads did not affect RPE proliferation (Fig. 4A). Interestingly, we observed the formation of multinucleate cells following POS treatment. Under standard culture conditions in the absence of POS, ~3% RPE cells were binucleate (Fig. 4B,F). The percentage of bi‐ and multinucleate RPE cells increased to 15% and 20% following POS and oxPOS treatment (Fig. 4C,F). Occasionally, cells with as many as 6 nuclei were observed in oxPOS‐treated cells (Fig. 4E). Furthermore, the size of each nucleus in multinucleate cells varied (Fig. 4C,E). oxPOS treatment also induced multinucleation in ARPE19 cells (data not shown). Interestingly, although latex beads did not affect RPE proliferation, exposure to and phagocytosis of latex beads for 48 h lead to around 10% bi‐/multinucleate RPE cell formation (Fig. 4D,F) indicating that these two processes were not directly interchangeable. Protein extracts from POS or oxPOS did not show any effects on RPE proliferation nor did they induce multinucleation (data not shown).

To mimic the *in vivo* situation of the aging RPE, in which focal defects in the monolayer may develop, we conducted the wound‐scratch assay in confluent ARPE19 cells. Under normal culture conditions, the wound healed within 3 days (Fig. S3A). OxPOS treatment significantly reduced the wound repair capacity of RPE cells (Fig. S3B,C). Furthermore, oxPOS treatment induced multinucleation in 4.5% of cells around the wound area, whereas <1% multinucleate cells were detected in the control group (Fig. S3D–F).

Both POS‐ and oxPOS‐induced suppression of RPE proliferation and multinucleation were reversible. When POS was removed from the culture, RPE cell proliferative activity was restored to normal levels while the number of multinucleate cells declined within 24 h (Fig. 4G). We did not detect significant number of apoptotic cells in POS‐ or oxPOS‐treated RPE cells, suggesting that the multinucleate cells may divide and become mononuclear cells after the removal of POS. As extracts of POS/oxPOS had no effect on suppression of RPE proliferation or on multinucleation, these data indicate that contact with POS/oxPOS is necessary to suppress RPE proliferation and induce multinucleation.

### Mechanism of multinucleate RPE cell formation

**Figure 5:**
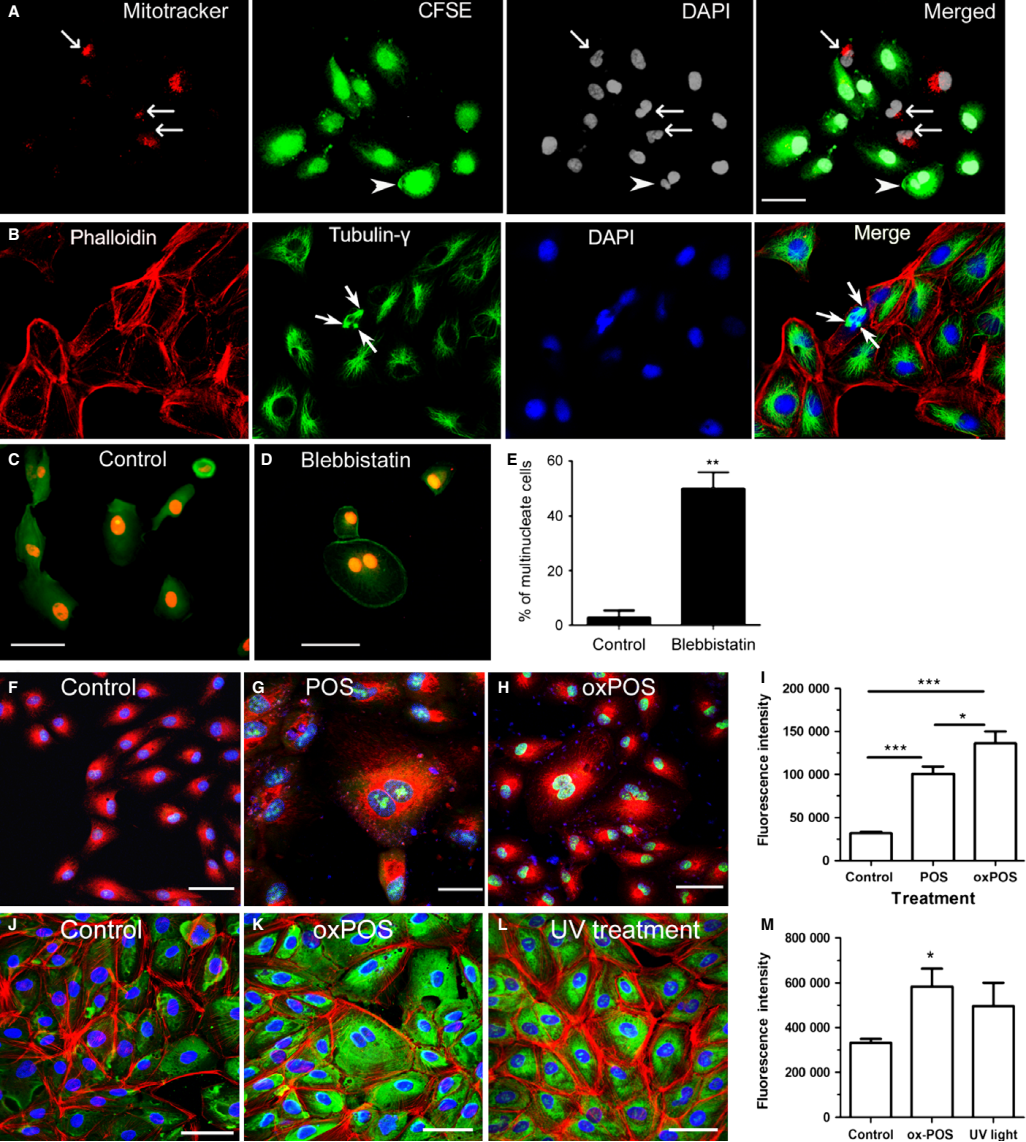
Mechanism of RPE multinucleation *in vitro*. (A) ARPE19 cells labelled with MitoTrackerRed or CFSE were mixed (1:1) and then treated with oxPOS (5:1) for 48 h, the cells were then fixed and stained for DAPI. Arrows indicate MitoTracker^+^ multinucleate RPE cells, and arrowhead indicates CFSE ^+^ multinucleate RPE cells. (B) ARPE19 cells were treated with oxPOS (5:1) for 48 h and stained for phalloidin, γ‐tubulin and DAPI. Arrows indicate a multinucleate cell with three centrosomes. (C) Control and blebbistatin‐treated ARPE19 cells were stained for phalloidin (green) and PI (red) and observed by confocal microscopy. 48% of blebbistatin‐treated cell were multinucleated. **, *P *< 0.01, unpaired Student's *t*‐test. *N *= 3. (F–H) CellRox Green staining in control (F), POS (G)‐ and oxPOS (H)‐treated ARPE19 cells. Green – CellRox Green, Red – MitoTracker, Blue – Hoechst 33342. I, Fluorescence intensity of CellRox Green in different groups of cells. (J–L) 8‐OHdG expression in control (J), oxPOS (K)‐ and UV light (L)‐ treated ARPE19 cells. Green – 8‐OHdG, red – phalloidin(F‐actin), blue – DAPI. M, Fluorescence intensity of 8‐OHdG in different groups of cells. *, *P *< 0.05; **, *P *< 0.01; ***, *P *< 0.001, *n *= 50 cells, Tukey's multiple comparison test.

The above results indicate that exposure to and/or phagocytosis of POS induces RPE cell multinucleation. However, whether multinucleation induced by POS was the result of phagocytosis of neighbouring RPE cells, fusion of neighbouring RPE cells or failed cytokinesis of RPE cells as a correlate of POS‐induced suppression of RPE cell proliferation remained unclear. To explore this process further, CFSE‐labelled ARPE19 cells were mixed with MitoTracker red‐labelled ARPE19 cells. The mixed cell cultures were then exposed to oxPOS for 48 h and the cells examined for single or dual fluorescence. Only single CFSE (arrowhead, Fig. 5A)‐ or MitoTracker Red (arrows, Fig. 5A)‐positive multinucleate RPE cells were observed. No dual‐positive cells were observed (Fig. 5A), suggesting that POS‐induced multinucleation was not caused by cell–cell fusion.

We next wished to address the possibility that RPE cell multinucleation occurred by failure of cytokinesis. The centrosome serves as the main microtubule organizing centre (MTOC) in mammalian cells as well as a regulator of cell cycle progression and is central to the process of cytokinesis. Gamma‐tubulin is essential for the nucleation and polar orientation of microtubules and is a critical component of the centrosome (Schiebel, 2000). A normal single nucleate cell has one centrosome, which can be copied once per cell cycle. However, when we examined POS‐treated RPE cells immunohistochemically using anti‐γ‐tubulin antibody, multiple centrosomes were observed in multinucleate cells (Fig. 5B), suggesting that a failure in cytokinesis underpinned the process of multinucleation in RPE cells.

We also evaluated the effect of direct pharmacological inhibition of cytokinesis on RPE multinucleation using blebbistatin, a potent myosin II inhibitor. Treatment of RPE cells with blebbistatin resulted in 48% multinucleate cells in ARPE19 cultures (Fig. 5C). To further understand whether oxidative stress is related to multiple centrosome formation and cytokinesis failure, intracellular reactive oxygen species (ROS) was measured using CellROX Green, a DNA‐binding fluorescence probe. POS treatment induced significant amount of ROS in ARPE19 cells, and oxPOS further enhanced ROS production (Fig. 5F–I). OxPOS‐induced DNA oxidation was further confirmed by 8‐OHdG staining (Fig. 5J–L).

### Phagocytic capacity of multinucleate RPE cells

As multinucleation rather than proliferation of RPE cells during aging appeared to be a mechanism whereby the RPE cell monolayer retained its integrity, it was important to determine whether multinucleate RPE cells remained functional. POS phagocytosis is one of the main functions of RPE cells, so we tested whether multinucleate RPE cells retained their phagocytic capability and remained healthy. Twelve‐month‐old mouse RPE eyecups were incubated with FITC‐labelled *E. Coli* BioParticles. Both multinucleate (arrowheads, Fig. S4A) and single nucleate (arrows, Fig. S4A) RPE cells were able to phagocytize *E. Coli* BioParticles. The amount of *E. Coli* BioParticles phagocytized by multinucleate cells appeared to be higher than single nucleate RPE cells (Fig. S4A). This was further confirmed in *in vitro* cultured ARPE19 cells (Fig. S4B,C). The average fluorescence intensity of phagocytized *E. Coli* per cells was significantly higher in multinucleate RPE cells compared to that in mononucleate cells (Fig. S4D). However, when the data were normalized by cell size (i.e. fluorescent intensity per μm^2^ of cell), there was no significant difference between RPE cells with one or more nucleus (Fig. S4E), suggesting a comparable level of phagocytic activity per μm^2^ of cell cytoplasmic area.

## Discussion

In this study, we find that RPE cells in the mouse eye show considerable morphological heterogeneity in shape and size depending on the location, becoming more marked from the central region of the eye towards the periphery, at all ages. In addition, the average RPE cell size increases with age and the cells become fewer in number as has been reported previously in human retina (Gao & Hollyfield, 1992). Interestingly, they become progressively multinucleate. In the central and equatorial regions, however, the number of nuclei per unit area remains the same; only in the peripheral region there is a commensurate reduction in RPE nuclei and thus an overall reduction in RPE cell number per unit area with age.

The integrity of the RPE cell monolayer is essential for normal functioning of the visual system, and indeed, a prominent feature of pathological aging in the retina is the development of areas of RPE atrophy, not only in AMD (Ardeljan & Chan, 2013; Mullins *et al*., 2014; Ozaki *et al*., 2014), but also in many forms of inherited retinal degeneration (McBain *et al*., 2012). Indeed, ‘dry’ AMD (geographic atrophy) is considered a form of apoptotic and/or necrotic cell death, possibly related to the loss of complement regulatory proteins (Ebrahimi *et al*., 2013; Hanus *et al*., 2013). As RPE cells *in vivo* are considered to be terminally differentiated, it is perhaps not surprising that with age, assuming even low levels of RPE cell loss (Gao & Hollyfield, 1992), the organism would respond by attempting to maintain the integrity of the RPE monolayer. The data reported here suggest that the RPE monolayer responds to age‐related cell loss, by increasing individual cell size and becoming multinucleate. Similar findings have been found in the marsupial RPE with age (quokka) (Fleming *et al*., 1996). Multinucleate RPE cells have also been reported to exist in human eyes (Starnes *et al*., ARVO 2015 Annual Meeting).

This raises the question as to how RPE cells become multinucleate with age. Multinucleate cells occur in several mammalian tissues, including human tissue, in both physiological and pathological conditions. Examples of physiological multinucleate cells are the many syncytia which occur in specialized tissues such as muscle (Abmayr & Pavlath, 2012) and in cells such as osteoclasts (Xing *et al*., 2012), while the formation of multinucleate giant cells (MGCs), of which there are several types (Brodbeck & Anderson, 2009), is a common feature of chronic inflammatory conditions (Anderson, 2000). In these conditions, multinucleation is considered to occur by cell fusion (Abmayr & Pavlath, 2012; Miyamoto, 2013), while multinucleation during development may occur by phagocytosis of live viable cells (Baluska *et al*., 2012). Recently, however, multinucleation in microglial cells was shown to occur by a mechanism involving failure of cytokinesis in which DNA synthesis and nuclear replication occurred, but progression to full cell division failed (Hornik *et al*., 2014). In the present study, we demonstrate *in vitro* that exposure to POS, particularly oxPOS, stimulated the formation of multinucleate RPE cells, and this appeared to occur by failure of cytokinesis rather than by cell fusion or phagocytosis of neighbouring RPE cells. Importantly, multinucleate RPE cells were functionally as active as single nucleate RPE cells, at least in terms of phagocytosis. However, *in vivo* some giant RPE cells, particularly those with diffused DNA staining, showed signs of cell damage, while cells containing pigment were observed on the surface of giant RPE cells in situ (Fig. 2B,C). We considered the latter cells to be either degenerate or dying RPE cells, or more probably, scavenging microglial cells which are known to survey the surface of the aging RPE and to engulf exocytosed melanin granules and other cell debris (Xu *et al*., 2008).

We investigated possible mechanisms whereby exposure of RPE cells to POS induced multinucleation and prevented cytokinesis in RPE cells. Phagocytosis of POS involves engagement of the actin–myosin cytoskeleton via the Mer/TK signalling complex whose activation controls the binding of POS to the RPE surface (Law *et al*., 2015) under the control of a recently reported regulatory gene, Klotho (Kokkinaki *et al*., 2013), while signalling via MerTK/Axl/Gas6 is required for phagocytosis of apoptotic cells in macrophages (Zagorska *et al*., 2014). However, the process of cytokinesis depends on much more extensive changes to the actomyosin/tubulin cytoarchitecture involving inhibition of centrosome polarization (Schiebel, 2000), and indeed, the close relationship between mutlinucleation and cytokinesis was confirmed in the present work where treatment with blebbistatin prevented cytokinesis in nearly 50% ARPE19 cells, and many cells became multinucleated. Therefore, interruption of the RPE phagocytic machinery was not a sufficient explanation. However, it is likely that the processes of POS phagocytosis and centrosome polarization intersect through multiple signalling pathways, study of which is outside the scope of this report, but merit further investigation.

An alternative explanation may lie in the observed suppression of RPE proliferation by exposure to POS (Fig. 4). Exposure of RPE cells to POS not only inhibited cytokinesis but also directly suppressed RPE proliferation. This effect was dose‐dependent, was stronger when the cells were exposed to oxPOS, and was not due to the process of phagocytosis as phagocytosis of latex beads had no effect on proliferative capacity of RPE cells. As mentioned above, RPE cells proliferate *in vitro* and have the potential to proliferate *in vivo* (Tosi *et al*., 2014). Loss of contact between the photoreceptors and the RPE seems to be the major stimulus for RPE migration and proliferation *in vivo* with progression to epithelial–mesenchyme transition (EMT) and the adoption of fibroblast characteristics with time (Chen *et al*., 2012). We show here soluble extracts of POS or oxPOS do not affect proliferation while contact with intact POS prevents RPE proliferation. This is confirmed by the resumption of proliferation after removal of the POS from the RPE monolayer (Fig. 4G). We propose therefore that under physiological conditions of health, POS prevents RPE proliferation through a contact‐mediated mechanism, that is a form of contact inhibition.

Contact inhibition is the *in vitro* correlate of terminal differentiation in cultured cells. Contact inhibition is a well‐recognized cell biological process whereby cell proliferation ceases when cultured cells come into contact as a confluent monolayer (Abercrombie, 1967) and is mediated through the Hippo signalling pathway involving the target transcription factors YAP/TAZ (Tariki *et al*., 2014) which are responsive to mechanical forces (Aragona *et al*., 2013). Sparse cultures of RPE cells *in vitro* proliferate extensively and adopt an EMT‐like spindle shape morphology (Newsome, 1983; Chen *et al*., 2012), but under appropriate conditions differentiate into a typical hexagonal nonproliferating RPE monolayer (Newsome, 1983). RPE cells *in vitro* also express Hippo‐related transcription factors when contact inhibited as a differentiated monolayer (Chen *et al*., 2012). The physiological signals by which POS induces differentiation and suppression of proliferation of RPE cells *in vitro* are not known, but the data in this study suggest that contact with the POS membrane or POS membrane‐associated molecular species derived from the interphotoreceptor matrix is necessary.

However, contact inhibition of RPE proliferation in itself does not provide a sufficient explanation for the processes of multinucleation and failure of cytokinesis observed here. Our further data suggest that oxidative stress may also be important. *In vitro* treatment of RPE cells with POS induced ROS production, and this was further enhanced by oxPOS. oxPOS also induced DNA oxidation. ROS‐mediated DNA damage is known to affect cell cycle and increase the risk of carcinogenesis (You & Bailis, 2010). oxPOS‐induced DNA oxidation may be one of the additional factors leading to multinucleation in RPE cells.

A unifying concept therefore may be that a major physiological function of POS may be to maintain homoeostatic integrity and the ‘postmitotic’ (terminally differentiated) phenotype of the RPE monolayer. With age however many factors, particularly oxidative stress, may compromise this physiological state as focal areas of RPE cell death appear. Neighbouring RPE cells enlarge and attempt to proliferate to repair the defect as in any epithelial monolayer. However, we propose that POS in partial contact with the enlarging/activated RPE cells continues to exert a suppressive effect on proliferation – the cells undergo some degree of DNA synthesis and even mitosis with nuclear replication but full cytokinesis and cell division is prevented by the POS. As a result, the aging RPE matures into a heterogeneous monolayer of different‐sized cells with multiple nuclei in which some of the very large cells may be moribund, but overall a functioning RPE monolayer is preserved. Evidence for this precarious balance can be seen in both experimental models with age (Chen *et al*., 2013; Kim *et al*., 2014) and in human retinal degenerative disease including AMD (Mullins *et al*., 2014; Ozaki *et al*., 2014).

**Figure 6:**
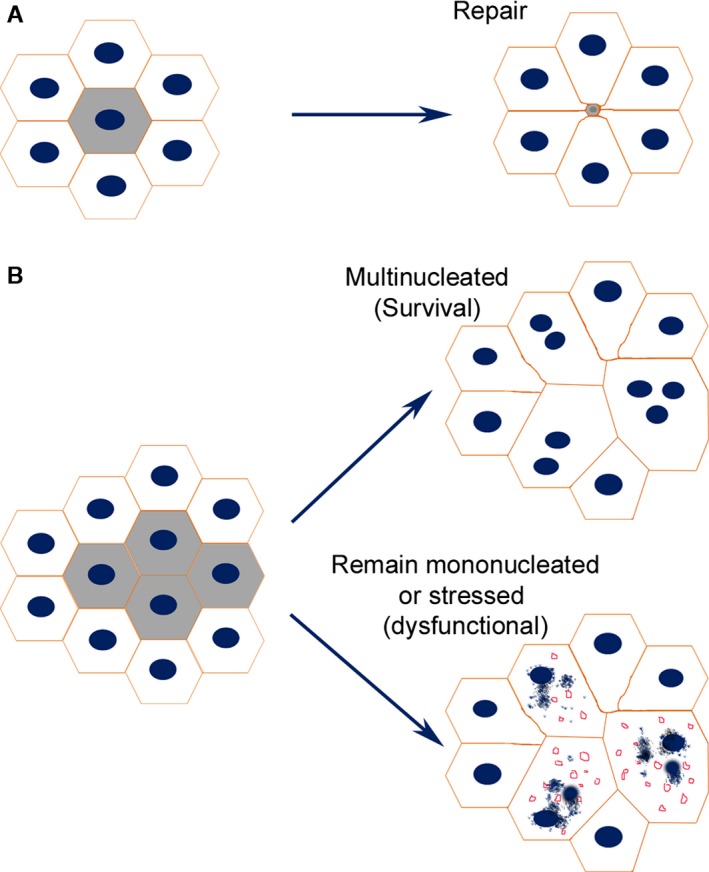
A Model of RPE cell repair during aging. (A) when a single RPE cell is damaged, adjacent cells expand in size and migrate towards the lesion site to repair the damage. (B) when many more cells are damaged over a sustained period of time, the remaining cells need to expand in size more extensively, sometimes up to 2–5 times of their original size to repair damage. The workload (e.g. phagocytize POS, transport nutrients and oxygen) of these enlarged hypertrophic RPE cells is 2–5 times more than their original workload. Multinucleated cell may cope with the substantial increase in cell volume and to maintain homoeostasis, but it is likely that this response is less efficient than cell replication. The stressed multinucleate cells or hypertrophic single nuclear cells may be at greater risk of cell death lead to patches of RPE loss (geographic atrophy).

We propose therefore that increase in cell size and multinucleation may be a strategy for the RPE cell monolayer to repair damage during aging (Fig. 6). It would appear that when RPE cell dies, the death signal may stimulate the remaining RPE cells to replicate its genetic material and increase in cell size to permit continued function of the RPE monolayer. However, this is a risky strategy as the potential for cell hypertrophy, even with multiple nuclei, is known to be limited (as for instance in muscle cells), and so the cell may be more prone to apoptotic cell death. Thus, patches of atrophy develop and present as areas of geographic atrophic or AMD in humans.

## Experimental procedures

### Animals

C57BL/6J mice were originally purchased from Harlan Laboratories (Blackthorn, UK) and bred either at the Biological Resource Unit at the Queen's University Belfast or the Medical Research Facility at the University of Aberdeen. All animals were housed in 12‐h light–dark cycle with free access to food and water. The *in vivo* protocols were approved by the Research Ethics Committee of the Queen's University Belfast and the University of Aberdeen, and all procedures conducted under the regulation of the UK Home Office Animals (Scientific Procedures) Act 1986 and were in compliance with the Association for Research in Vision and Ophthalmology Statement for the Use of Animals in Ophthalmology and Vision Research.

### Preparation of RPE flat mounts

The eyes were collected from mice of different ages and fixed with 2% paraformaldehyde for 2 h. The anterior segment (cornea, iris and ciliary body, and lens) of the eye was removed. Four to five radical cuts were made from the edge of the eyecup to the equator. Retinal tissue was carefully removed from the eyecup, and the remaining cups containing RPE, choroid and sclera were thoroughly washed and processed for immunofluorescence staining.

### *In vitro* RPE cell culture

Primary mouse RPE cells were cultured from 3‐month‐old C56BL/6J mice as described previously (Chen *et al*., 2008; Luo *et al*., 2011). Briefly, after removing the anterior segment of the eye, the vitreous and retina, the RPE/choroid/sclera eyecups, were incubated with 0.5% (w/v) trypsin–EDTA (ICN Flow, Irvin, UK) at 37°C for 30 min. RPE single‐cell suspension was collected and seeded into culture plates with complete DMEM (DMEM supplemented with 10% FCS, 100 μg mL^−1^ primocin). Cells were subcultured when they reached confluence. The phenotype of RPE cells was confirmed by RPE65 immunostaining at passage. Only cells with >95% purity were used in the study. Passage 3–5 cells were used for experiments.

The B6‐RPE07 mouse RPE cell was cultured in DMEM with 10% FCS as previous described (Chen *et al*., 2008). Cells were passaged at a ratio of 1:4 once a week. Human ARPE19 cells (originally purchased from ATCC, CRL‐2302, Middlesex, UK) were cultured in DMEM/F12 (Life Technologies Ltd, Paisley, UK) supplemented with 15% FCS and subcultured at a ratio of 1:3 once every week.

### Preparation of photoreceptor outer segments

Photoreceptor outer segments (POS) were isolated from bovine eyes using the sucrose gradient density centrifugation as previous described (Chen *et al*., 2007). Oxidized POS (ox‐POS) was generated by exposing POS to 302 nm ultraviolet light for 18 h as described previously (Chen *et al*., 2007), and lipid oxidation was confirmed by the thiobarbituric acid reactive substance assay kit (Alexis; Axxora Ltd, Nottingham, UK).

### *In vitro* proliferation assay

Primary mouse RPE cells or B6‐RPE07 cells were seeded in 96‐well plates (3 × 10^3^ cells/well in 200 μL) and incubated at 37°C 5% CO2 for 16 h. The cells were then treated with different concentrations of POS or latex beads (1.1 μm; Sigma‐Aldrich, Dorset, UK) (POS: RPE ratio = 1:1; 5:1; 10:1) for 48 h. Cell proliferation was measured by MTT assay following manufacturer's protocol.

### *In vitro* generation of multinucleate RPE cells

Primary mouse RPE cells, B6‐RPE07 cells or ARPE19 (5 × 10^2^) cells were seeded on 20 mm coverslips in a 12‐well plate and incubated for 16 h. The cells were then treated with POS, ox‐POS or latex beads for 48 h (POS:RPE** = **5:1). The cells (in coverslip) were then washed and fixed with methanol (Agar Scientific Ltd., Cambridge, UK) at −20**°**C for 30 min and processed further for immunostaining.

To induce multinucleate RPE cells by cytokinesis disruption, ARPE19 cells were treated with 0.2 μg mL^−1^ nocodazole (Abcam, Cambridge, UK) for 16 h. Mitotic cells were detached by shaking the culture dishes. The mitotic cells were then cultured on cover slips in a 12‐well plate at a density of 1 × 10^3^ per well with the myosin II ATPase inhibitor, blebbistatin (50 mmol) (Abcam) for 22 h. Cells were then washed thoroughly to remove blebbistatin and cultured for a further 8 h before sampling for immunostaining.

### *In vivo* BrdU labelling

Proliferative activity of RPE *in vivo* was determined by bromodeoxyuridine (5‐bromo‐2′‐deoxyuridine, BrdU) labelling as follows: 3‐ and 12‐month‐old C57BL/6J mice (*n***** **= **6 in each group) were injected with 100 μg g^**−**1^ body weight BrdU (Sigma‐Aldrich) intraperitoneally once a day for 7 days. The animals were then sacrificed by CO_2_ inhalation. Eyes were collected for RPE/choroid/sclera flat mount staining using anti‐BrdU antibody.

### RPE fusion study

ARPE19 cells were incubated with 5 μm mL^−1^ CFSE or 100 nm mL^−1^ MitoTrackerRed (both from Life Technologies, Carlsbad, CA, USA) for 15 min at 37°C. After thorough washes with PBS, 500 CFSE‐labelled cells were mixed with 500 MitoTracker‐labelled cells and seeded on coverslips in a 12‐well plate, and incubated for 6 h at 37°C. Cells were then treated with 1 × 10^6 ^mL^−1^ oxPOS for 48 h. The coverslips were washed with PBS and fixed with 2% PFA for 15 min at room temperature. The coverslips were mounted with antifade Vectashield medium with DAPI (Vector Lab Ltd. Peterborough, UK) on glass slides and observed by confocal microscopy (Eclipse TE2000‐U; Nikon UK Ltd, Surrey, UK).

### Immunofluorescence staining and confocal microscopy

RPE/choroid/sclera tissues were permeabilized with 1% Triton X‐100 at room temperature for 2 h. The samples were then incubated with FITC‐conjugated anti‐mouse α‐tubulin (1:50,) Alexa Fluor^R^ 568 or Alexa Fluor^R^ 488 Phalloidin (1:100; Life Technologies) and propidium iodide (PI, 1:100, Sigma‐Aldrich) or 4′,6‐diamidino‐2‐phenylindole (DAPI, 1:100 Sigma‐Aldrich) or FITC‐conjugated anti‐human BrdU antibody (1:50; Cymbus Biotechnology, Eastleigh, Hampshire, UK) at 4°C for overnight. After thorough washes, samples were flat mounted on glass slides with RPE layer face up using antifade Vectashield medium (Vector Lab Ltd). All samples were examined using a confocal laser microscope (Zeiss LSM510, Zeiss, or Eclipse TE2000‐U).

RPE cells in coverslips were permeabilized with 1% Triton X‐100 for 5 min at 4°C. The samples were then incubated with primary antibodies for 4 h followed by FITC‐conjugated second antibody (goat anti‐mouse IgG, 1:100) or Alexa Fluor^R^ 568 Phalloidin (1:100, both from Life Technologies) for 1 h. Primary antibodies used include mouse anti‐RPE65 (1:50; Novus Biologicals, Littleton, CO, USA), mouse anti‐γ‐tubulin (Life Technologies) and mouse anti‐8 OHdG (Abcam). In addition, FITC‐conjugated anti‐mouse α‐tubulin (1:50; Sigma‐Aldrich) was also used in one‐step immunostaining. The coverslips were then mounted on glass slides with Vectashield medium with DAPI (Vector Lab Ltd) and observed by confocal microscopy.

### Detection of reactive oxygen species (ROS) using CellRox Green

ARPE19 cells cultured in 12‐well places with/without POS or oxPOS were treated with 5 μm well^−1^ CellRox Green (Life Technologies) for 30 min, followed by Mito Tracker Red (Life Technologies) and Hoechst 33342 (Thermo Fisher Scientific Loughborough, UK) for a further 30 min. After thorough washes, fresh medium was added to the well and cells imaged by confocal microscopy. The fluorescence intensity of CellRox green in each cell and background fluorescence from cell‐free area was measured using ImageJ software 1.45. The background fluorescence intensity was deducted from the CellRox green intensity of cells for data analysis.

### Image analysis

RPE/choroid/sclera flat mounts were divided into central, equatorial and peripheral regions (Fig. 1A). The distance from the inner edge to the outer edge within the region is the same in the three regions (Fig. 1A). Five z‐stack images were taken from each region. The z‐stack confocal images were then reconstructed using the LSM Imaging Browser (Zeiss) system. The margin of RPE cell was identified by phalloidin staining, and the size of each RPE cell was measured manually using the same software. RPE nucleus and cell numbers were counted automatically using the Volocity. The numbers of cell/nucleus from 5 images of the same region were averaged, and the averaged number was considered as the number of cell/nucleus of that region of the sample.

The number of binucleate or multinucleate RPE cells in cell cultures was counted manually using the confocal microscope (Eclipse TE2000‐U). A total of 200 cells from each coverslip and three coverslips from each group were counted.

## Funding

The study was funded by Fight for Sight (1361/1362, 1425/1426) and the Development Trust of the University of Aberdeen. Dr Heping Xu was a Research Council UK (2005‐2009) (RCUK) academic fellow funded by the Department of Trade and Industry and Office of Science and Technology.

## Conflict of interest

None declared.

## Author contributions

MC performed most of the *in vivo* and *in vitro* experiments. HX, MC and JVF designed the study, interpreted the results and wrote the manuscript. DR performed part of the *in vitro* studies, and MF performed part of the RPE flat mount staining and image analysis. CL performed the eyecup phagocytosis assay.

## Supporting information


**Fig. S1** Mononucleate, binucleate and multinucleate RPE cells in mice of different ages.

**Fig. S2** RPE cell size in mice of different ages.

**Fig. S3** The effect of oxPOS on RPE cell wound healing.

**Fig. S4** RPE cell phagocytosis ex vivo and *in vitro*.

**Data S1** Materials and methods.

## References

[acel12447-bib-0001] Abercrombie M (1967) Contact inhibition: the phenomenon and its biological implications. Natl. Cancer Inst. Monogr. 26, 249–277. 4864106

[acel12447-bib-0002] Abmayr SM , Pavlath GK (2012) Myoblast fusion: lessons from flies and mice. Development 139, 641–656. 22274696 10.1242/dev.068353 PMC3265056

[acel12447-bib-0003] Anderson JM (2000) Multinucleated giant cells. Curr. Opin. Hematol. 7, 40–47. 10608503 10.1097/00062752-200001000-00008

[acel12447-bib-0004] Aragona M , Panciera T , Manfrin A , Giulitti S , Michielin F , Elvassore N , Dupont S , Piccolo S (2013) A mechanical checkpoint controls multicellular growth through YAP/TAZ regulation by actin‐processing factors. Cell 154, 1047–1059. 23954413 10.1016/j.cell.2013.07.042

[acel12447-bib-0005] Ardeljan D , Chan CC (2013) Aging is not a disease: distinguishing age‐related macular degeneration from aging. Prog. Retin. Eye Res. 37, 68–89. 23933169 10.1016/j.preteyeres.2013.07.003 PMC3830684

[acel12447-bib-0006] Baluska F , Volkmann D , Menzel D , Barlow P (2012) Strasburger's legacy to mitosis and cytokinesis and its relevance for the Cell Theory. Protoplasma 249, 1151–1162. 22526203 10.1007/s00709-012-0404-8

[acel12447-bib-0007] Brodbeck WG , Anderson JM (2009) Giant cell formation and function. Curr. Opin. Hematol. 16, 53–57. 19057205 10.1097/MOH.0b013e32831ac52e PMC2679387

[acel12447-bib-0008] Chen M , Forrester JV , Xu H (2007) Synthesis of complement factor H by retinal pigment epithelial cells is down‐regulated by oxidized photoreceptor outer segments. Exp. Eye Res. 84, 635–645. 17292886 10.1016/j.exer.2006.11.015

[acel12447-bib-0009] Chen M , Muckersie E , Robertson M , Fraczek M , Forrester JV , Xu H (2008) Characterization of a spontaneous mouse retinal pigment epithelial cell line B6‐RPE07. Invest. Ophthalmol. Vis. Sci. 49, 3699–3706. 18421091 10.1167/iovs.07-1522

[acel12447-bib-0010] Chen HC , Zhu YT , Chen SY , Tseng SC (2012) Wnt signaling induces epithelial‐mesenchymal transition with proliferation in ARPE‐19 cells upon loss of contact inhibition. Lab. Invest. 92, 676–687. 22391957 10.1038/labinvest.2011.201 PMC3961713

[acel12447-bib-0011] Chen M , Hombrebueno JR , Luo C , Penalva R , Zhao J , Colhoun L , Pandi SP , Forrester JV , Xu H (2013) Age‐ and Light‐Dependent Development of Localised Retinal Atrophy in CCL2(‐/‐)CX3CR1(GFP/GFP) Mice. PLoS ONE 8, e61381. 23637822 10.1371/journal.pone.0061381 PMC3630229

[acel12447-bib-0012] Ebrahimi KB , Fijalkowski N , Cano M , Handa JT (2013) Decreased membrane complement regulators in the retinal pigmented epithelium contributes to age‐related macular degeneration. J. Pathol. 229, 729–742. 23097248 10.1002/path.4128 PMC3836183

[acel12447-bib-0013] Fleming PA , Harman AM , Beazley LD (1996) Retinal pigment epithelium topography in the mature quokka, *Setonix brachyurus* . Exp. Eye Res. 62, 85–93. 8674516 10.1006/exer.1996.0010

[acel12447-bib-0014] Gao H , Hollyfield JG (1992) Aging of the human retina. Differential loss of neurons and retinal pigment epithelial cells. Invest. Ophthalmol. Vis. Sci. 33, 1–17. 1730530

[acel12447-bib-0015] Hanus J , Zhang H , Wang Z , Liu Q , Zhou Q , Wang S (2013) Induction of necrotic cell death by oxidative stress in retinal pigment epithelial cells. Cell Death Dis. 4, e965. 24336085 10.1038/cddis.2013.478 PMC3877549

[acel12447-bib-0016] Hesse M , Raulf A , Pilz GA , Haberlandt C , Klein AM , Jabs R , Zaehres H , Fugemann CJ , Zimmermann K , Trebicka J , Welz A , Pfeifer A , Roll W , Kotlikoff MI , Steinhauser C , Gotz M , Scholer HR , Fleischmann BK (2012) Direct visualization of cell division using high‐resolution imaging of M‐phase of the cell cycle. Nat. Commun. 3, 1076. 23011130 10.1038/ncomms2089 PMC3658003

[acel12447-bib-0017] Hornik TC , Neniskyte U , Brown GC (2014) Inflammation induces multinucleation of Microglia via PKC inhibition of cytokinesis, generating highly phagocytic multinucleated giant cells. J. Neurochem. 128, 650–661. 24117490 10.1111/jnc.12477

[acel12447-bib-0018] Kim SY , Yang HJ , Chang YS , Kim JW , Brooks M , Chew EY , Wong WT , Fariss RN , Rachel RA , Cogliati T , Qian H , Swaroop A (2014) Deletion of aryl hydrocarbon receptor AHR in mice leads to subretinal accumulation of microglia and RPE atrophy. Invest. Ophthalmol. Vis. Sci. 55, 6031–6040. 25159211 10.1167/iovs.14-15091 PMC4176417

[acel12447-bib-0019] Kokkinaki M , Abu‐Asab M , Gunawardena N , Ahern G , Javidnia M , Young J , Golestaneh N (2013) Klotho regulates retinal pigment epithelial functions and protects against oxidative stress. J. Neurosci. 33, 16346–16359. 24107965 10.1523/JNEUROSCI.0402-13.2013 PMC3810551

[acel12447-bib-0020] Law AL , Parinot C , Chatagnon J , Gravez B , Sahel JA , Bhattacharya SS , Nandrot EF (2015) Cleavage of Mer Tyrosine Kinase (MerTK) from the cell surface contributes to the regulation of retinal phagocytosis. J. Biol. Chem. 290, 4941–4952. 25538233 10.1074/jbc.M114.628297 PMC4335232

[acel12447-bib-0021] Lee TT , Martin FC , Merrill JE (1993) Lymphokine induction of rat microglia multinucleated giant cell formation. Glia 8, 51–61. 8509164 10.1002/glia.440080107

[acel12447-bib-0022] Lim LS , Mitchell P , Seddon JM , Holz FG , Wong TY (2012) Age‐related macular degeneration. Lancet 379, 1728–1738. 22559899 10.1016/S0140-6736(12)60282-7

[acel12447-bib-0023] Luo C , Chen M , Xu H (2011) Complement gene expression and regulation in mouse retina and retinal pigment epithelium/choroid. Mol. Vis. 17, 1588–1597. 21738388 PMC3123163

[acel12447-bib-0024] MacLauchlan S , Skokos EA , Meznarich N , Zhu DH , Raoof S , Shipley JM , Senior RM , Bornstein P , Kyriakides TR (2009) Macrophage fusion, giant cell formation, and the foreign body response require matrix metalloproteinase 9. J. Leukoc. Biol. 85, 617–626. 19141565 10.1189/jlb.1008588 PMC2656428

[acel12447-bib-0025] McBain VA , Townend J , Lois N (2012) Progression of retinal pigment epithelial atrophy in stargardt disease. Am. J. Ophthalmol. 154, 146–154. 22464366 10.1016/j.ajo.2012.01.019

[acel12447-bib-0026] Miyamoto T (2013) STATs and macrophage fusion. JAKSTAT 2, e24777. 24069561 10.4161/jkst.24777 PMC3772113

[acel12447-bib-0027] Mullins RF , Khanna A , Schoo DP , Tucker BA , Sohn EH , Drack AV , Stone EM (2014) Is age‐related macular degeneration a microvascular disease? Adv. Exp. Med. Biol. 801, 283–289. 24664709 10.1007/978-1-4614-3209-8_36 PMC8412173

[acel12447-bib-0028] Newsome DA (1983) Retinal pigmented epithelium culture: current applications. Trans. Ophthalmol. Soc. UK 103(Pt 4), 458–466. 6380009

[acel12447-bib-0029] Nussenblatt RB , Lee RW , Chew E , Wei L , Liu B , Sen HN , Dick AD , Ferris FL (2014) Immune responses in age‐related macular degeneration and a possible long‐term therapeutic strategy for prevention. Am. J. Ophthalmol. 158, 5–11.e2. 24709810 10.1016/j.ajo.2014.03.014 PMC4058353

[acel12447-bib-0030] Oh Y , Oh I , Morimoto J , Uede T , Morimoto A (2014) Osteopontin has a crucial role in osteoclast‐like multinucleated giant cell formation. J. Cell. Biochem. 115, 585–595. 24129963 10.1002/jcb.24695

[acel12447-bib-0031] Ozaki E , Campbell M , Kiang AS , Humphries M , Doyle SL , Humphries P (2014) Inflammation in age‐related macular degeneration. Adv. Exp. Med. Biol. 801, 229–235. 24664703 10.1007/978-1-4614-3209-8_30

[acel12447-bib-0032] Park JK , Askin F (2013) Osteoclast‐like multinucleated giant cells in sinonasal inflammation of granulomatosis with polyangiitis (Wegener's granulomatosis). Clin. Exp. Rheumatol. 31, S28–S31. 23465045

[acel12447-bib-0033] Pfeffer BA , Philp NJ (2014) Cell culture of retinal pigment epithelium: Special Issue. Exp. Eye Res. 126, 1–4. 25152358 10.1016/j.exer.2014.07.010

[acel12447-bib-0034] Schiebel E (2000) Gamma‐tubulin complexes: binding to the centrosome, regulation and microtubule nucleation. Curr. Opin. Cell Biol. 12, 113–118. 10679351 10.1016/s0955-0674(99)00064-2

[acel12447-bib-0035] Strauss O (2005) The retinal pigment epithelium in visual function. Physiol. Rev. 85, 845–881. 15987797 10.1152/physrev.00021.2004

[acel12447-bib-0036] Tariki M , Dhanyamraju PK , Fendrich V , Borggrefe T , Feldmann G , Lauth M (2014) The Yes‐associated protein controls the cell density regulation of Hedgehog signaling. Oncogenesis 3, e112. 25111861 10.1038/oncsis.2014.27 PMC5189961

[acel12447-bib-0037] Tosi GM , Marigliani D , Romeo N , Toti P (2014) Disease pathways in proliferative vitreoretinopathy: an ongoing challenge. J. Cell. Physiol. 229, 1577–1583. 24604697 10.1002/jcp.24606

[acel12447-bib-0038] Ts'o MO , Friedman E (1967) The retinal pigment epithelium. I. Comparative histology. Arch. Ophthalmol. 78, 641–649. 4963693 10.1001/archopht.1967.00980030643016

[acel12447-bib-0039] Ts'o MO , Friedman E (1968) The retinal pigment epithelium. 3. Growth and development. Arch. Ophthalmol. 80, 214–216. 5661888 10.1001/archopht.1968.00980050216012

[acel12447-bib-0040] Vignery A (2005) Macrophage fusion: are somatic and cancer cells possible partners? Trends Cell Biol. 15, 188–193. 15817374 10.1016/j.tcb.2005.02.008

[acel12447-bib-0041] Xing L , Xiu Y , Boyce BF (2012) Osteoclast fusion and regulation by RANKL‐dependent and independent factors. World J. Orthop. 3, 212–222. 23362465 10.5312/wjo.v3.i12.212 PMC3557323

[acel12447-bib-0042] Xu H , Chen M , Manivannan A , Lois N , Forrester JV (2008) Age‐dependent accumulation of lipofuscin in perivascular and subretinal microglia in experimental mice. Aging Cell 7, 58–68. 17988243 10.1111/j.1474-9726.2007.00351.x

[acel12447-bib-0043] You Z , Bailis JM (2010) DNA damage and decisions: CtIP coordinates DNA repair and cell cycle checkpoints. Trends Cell Biol. 20, 402–409. 20444606 10.1016/j.tcb.2010.04.002 PMC5640159

[acel12447-bib-0044] Zagorska A , Traves PG , Lew ED , Dransfield I , Lemke G (2014) Diversification of TAM receptor tyrosine kinase function. Nat. Immunol. 15, 920–928. 25194421 10.1038/ni.2986 PMC4169336

