# Supplementary figures and images for: Retinal pigment epithelial cell multinucleation in the aging eye – a mechanism to repair damage and maintain homoeostasis
Source: Aging Cell. 2016 Feb 15;15(3):436–45. doi: 10.1111/acel.12447 (PMC4854907; doi:10.1111/acel.12447)

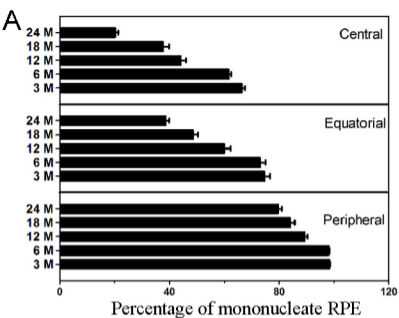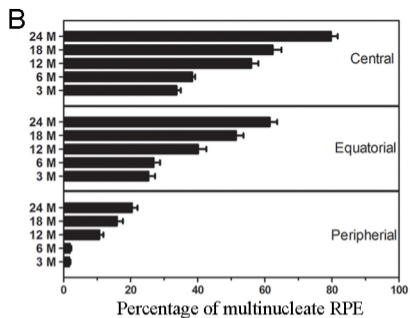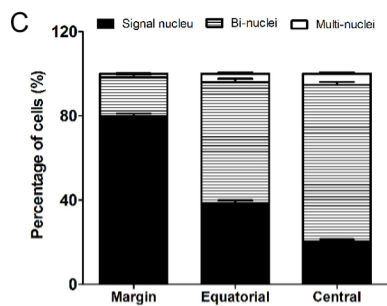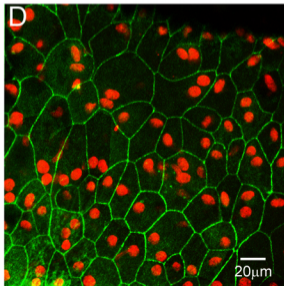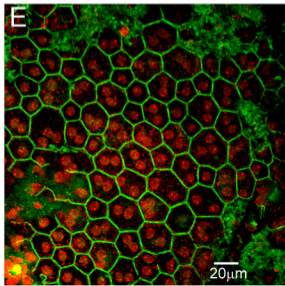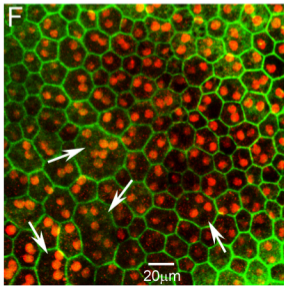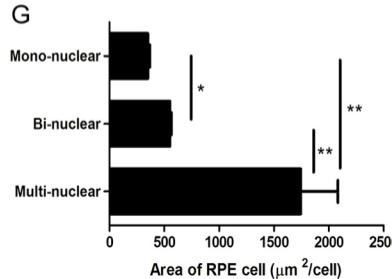

Supplement: Supplementary file 1 — Fig. S1 Mononucleate, binucleate and multinucleate RPE cells in mice of different ages. [file ACEL-15-436-s001.pdf]

A

Central

Equatorial

Peripheral

3 Months

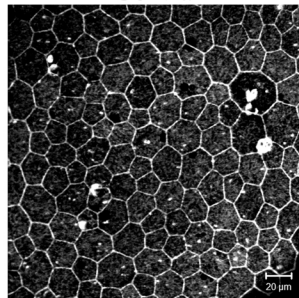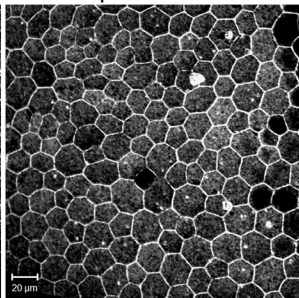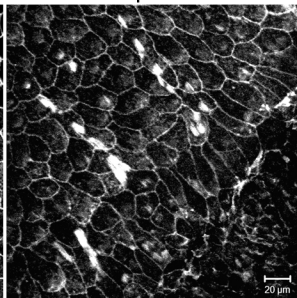

12 Months

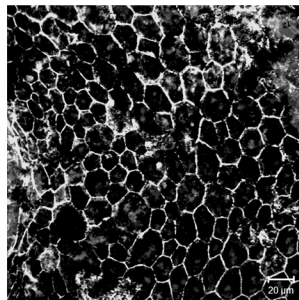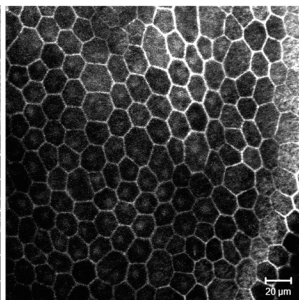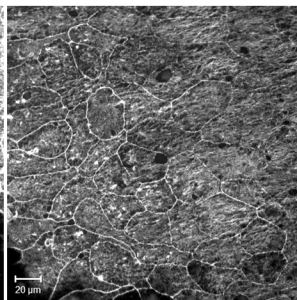

24 Months

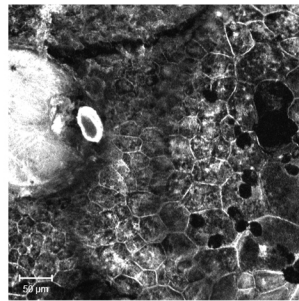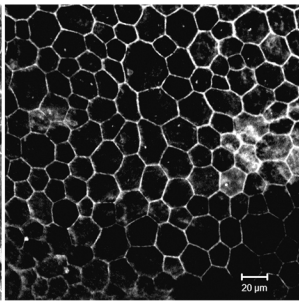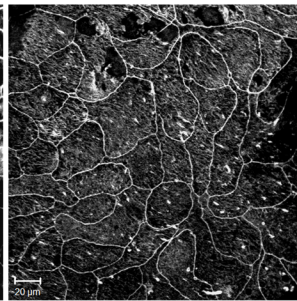

B

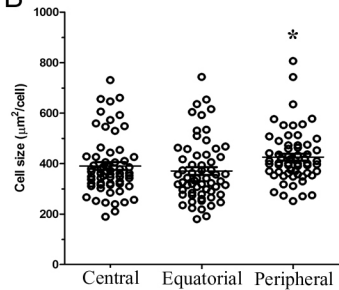

C

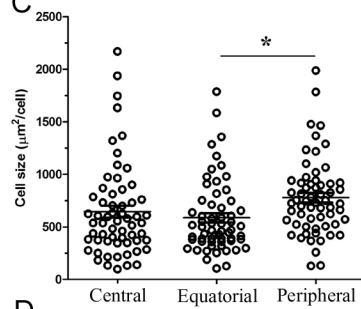

D

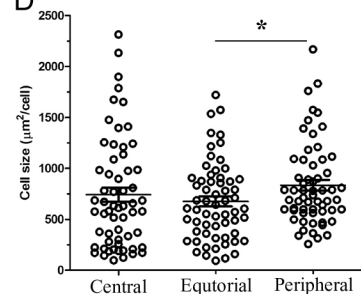

Supplement: Supplementary file 2 — Fig. S2 RPE cell size in mice of different ages. [file ACEL-15-436-s002.pdf]

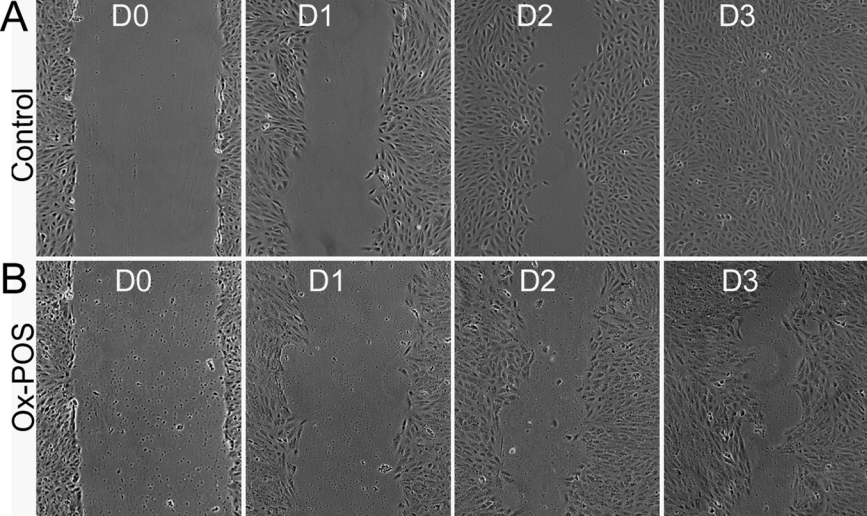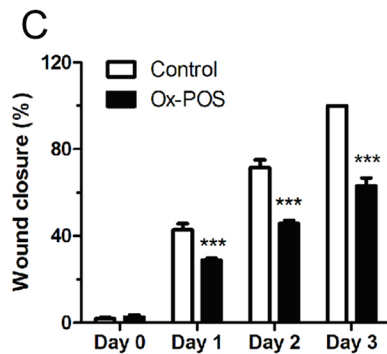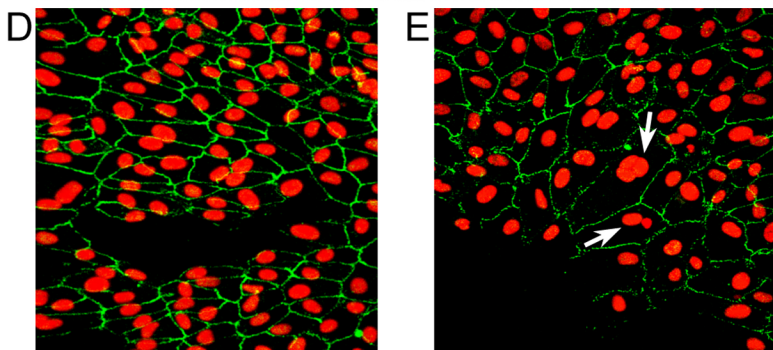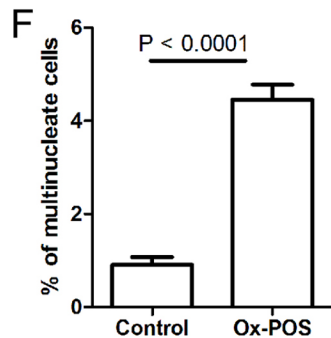

Supplement: Supplementary file 3 — Fig. S3 The effect of oxPOS on RPE cell wound healing. [file ACEL-15-436-s003.pdf]

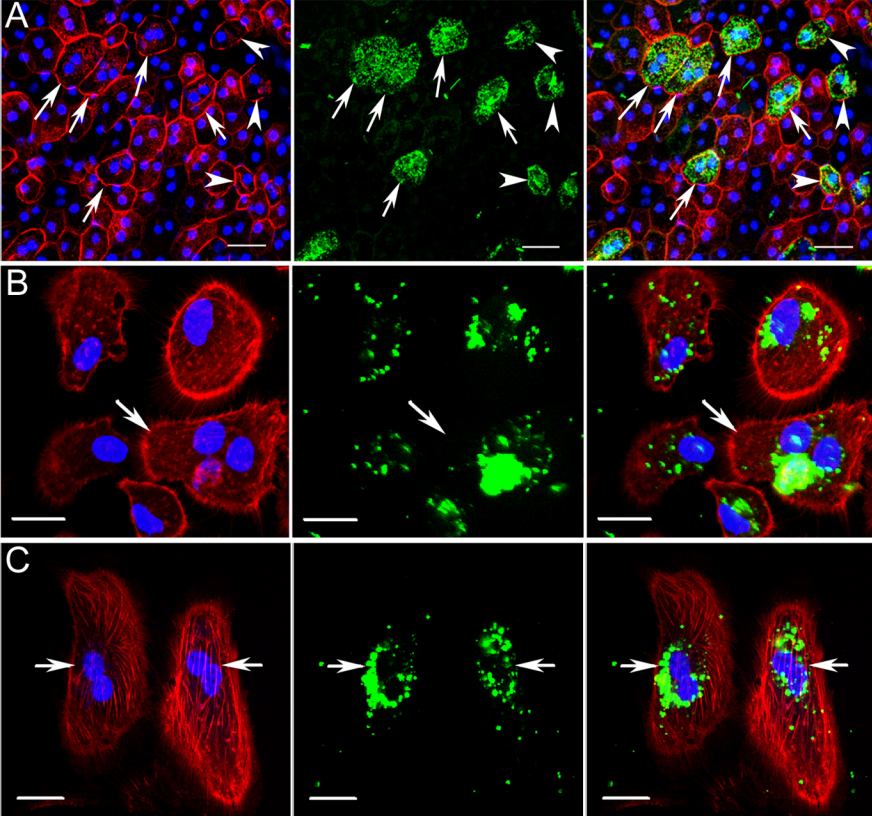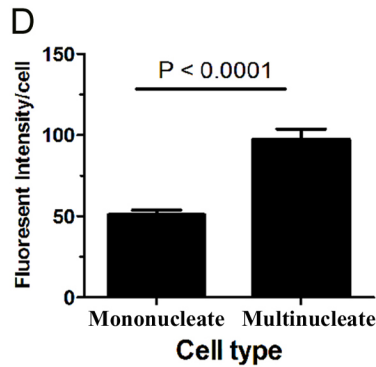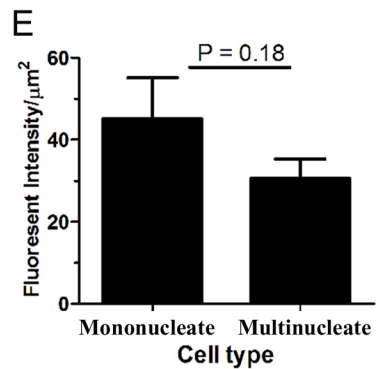

Supplement: Supplementary file 4 — Fig. S4 RPE cell phagocytosis ex vivo and in vitro. [file ACEL-15-436-s004.pdf]
