# Supplementary material for: Retinal pigment epithelial cell multinucleation in the aging eye – a mechanism to repair damage and maintain homoeostasis
Source: Aging Cell. 2016 Feb 15;15(3):436–45. doi: 10.1111/acel.12447 (PMC4854907; doi:10.1111/acel.12447)
Supplement: Supplementary file 5 — Data S1 Materials and methods. [file ACEL-15-436-s005.docx]

**Supplementary Materials and Methods**

**Wound healing experiment**

ARPE-19 cells were cultured on 12-well plates with complete medium (DMEM-F12 and 10% Foetal calf serum) at 37ºC for until confluent. OxPOS (1ˣ10^6^ particles/ml) was added into eight wells and another eight wells were used as controls. 24 h later, a P200 pipette tip was used to create a uniform single scratch in each well. Fresh ox-POS was added to the treatment wells daily. Five images at 5 different scratch locations were taken from each well on days 0, 1, 2 and 3. The average wound area for each well was calculated using Image J software version 1.45.

At day two, four control and four ox-POS treated wells were fixed with 2% PFA for 15 min. The cells were then washed with PBSA and permeabilised using 1% Triton X for 5 min. The cells were then incubated with rabbit anti-ZO-1(1:100, ThermoFisher Scientific, Loughborough, UK) for 2 h, followed by FITC conjugated goat anti-rabbit IgG and DAPI nuclear staining (ThermoFisher Scientific). Five images were obtained from different locations of each well using confocal microscopy (Eclipse TE2000-U). The percentage of bi-, multi-nuclear cells was analysed using Image J software version 1.45.

### Phagocytosis assay

Multinucleate RPE cells were induced by exposing the cells to ox-POS as described above. Forty-eight hours after ox-POS treatment, cells were washed thoroughly to remove excessive POS. FITC-conjugated E.Coli bioparticles (Vybrant Phagocytosis Assay Kit, V-6694, Life Technologies) were added to each well at a 1:20 ratio of cell to bioparticles. After 12h incubation, the cells were washed and fixed with 2% PFA for 15 min. The samples were further stained for DAPI (1:50) and F-actin using Phalloidin-iFluor 555 (1:50, Abcam). All samples were imaged by confocal microscopy (Eclipse TE2000-U).

The fluorescence intensity of E.Coli particles in mononucleate, binucleate and multinucleate RPE cells were measured using the public domain free software Image J system. A total of 100 cells from each cell type (mononucleate, bi-, multi-nucleate) were counted from each coverslip and three coverslips were included in each treatment group.
