# Supplementary material for: Retinal pigment epithelial cell multinucleation in the aging eye – a mechanism to repair damage and maintain homoeostasis
Source: Aging Cell. 2016 Feb 15;15(3):436–45. doi: 10.1111/acel.12447 (PMC4854907; doi:10.1111/acel.12447)
Supplement: Supplementary file 6 [file ACEL-15-436-s006.docx]

**Manuscript ID: ACE-15-0315**

**Title: Retinal pigment epithelial cell multinucleation in the aging eye – a mechanism to repair damage and maintain homeostasis**

**Authors:** Mei Chen, Dinusha Rajapakse, Monika Fraczek, Chang Luo, John V Forrester, Heping Xu

**Supplementary figure legends**

**Supplement Figure 1**. **Mononucleate, binucleate and multinucleate RPE cells in mice of different ages.** RPE/choroid/sclera flatmounts were stained with phalloidin (green) and PI (red) and imaged by confocal microscopy. A, the percentage of mononucleate RPE cells in the central, equatorial and peripheral regions in different ages of mice. B, the percentage of bi-/multi-nucleate RPE cells in the central, equatorial and peripheral regions in different ages of mice. C, the percentage of single nucleus, bi-nuclei and multi-nucleate RPE cells in the central, equatorial and peripheral regions in 24 months old mice. D-F, typical confocal images of RPE flatmount from a 24m old mouse showing RPE cells in the peripheral (D), equatorial (E) and central (F) regions. G, the average size of mononucleate, bi-nucleate and multinucleate RPE cells from the central region of 24 months old mice. *, P<0.05; **, P<0.01. One way ANOVA followed by Tukey’s multiple comparison test. N ≥60.

**Supplement Figure 2**. **RPE cell size in mice of different ages.** RPE/choroid/sclera flatmounts from 3, 12 and 24 months old mice were stained with phalloidin and imaged by confocal microscopy. A, typical confocal images of RPE cells in the peripheral, equatorial and central regions. B-D, the size of RPE cells in different regions of the eye from 3 months (B), 12 months (C) and 24 months (D) old mice. *, P<0.05 , One way ANOVA followed by Tukey’s multiple comparison test. N = 60.

**Supplement Figure 3**. The effect of oxPOS on RPE cell wound healing. Confluent ARPE19 cells were treated with/without 1x10^6^oxPOS/ml for 24h. A wound scratch was made to the cell monolayer. Fresh oxPOS was added to the treatment group each day. A & B, representative phase-contrast images taken from each group on different days. C, bar figure showing the percentage of wound closure at different days from different groups. ***, P < 0.001 compared to the control group of the same day. N = 8. D & E, representative confocal images taken from the wound area of the control (D) and oxPOS treated (E) cell stained for PI and ZO-1 (Green) at 48h. F, bar figure showing the percentage of bi-, multi-nucleated cells in each group at 48h.

**Supplement Figure 4. RPE cell phagocytosis ex vivo and in vitro.** A, RPE/choroid/sclera eyecups from 12-months old mice were incubated with FITC-E.Coli BioParticles for 6h. The flatmounts were stained with phalloidin (red) and DAPI (blue) and imaged by confocal microscopy. Arrows indicating multinucleate RPE cells and arrowhead indicating mononucleate RPE cells. B and C, ARPE19 cells were treated with oxPOS (5:1) for 48h, the cells were then washed and incubated with FITC-E.Coli for 12h. Cells were then fixed and stained for DAPI and F-actin and imaged by confocal microscopy. D, histogram shown the average fluorescent intensity of E.Coli in mononucleate and multinucleate RPE cells as expressed “Fluorescent intensity/cell”. E, histogram showing the average fluorescent intensity of E.Coli in mononucleate and multinucleate RPE cells as expressed “Fluorescent intensity/μm^2^ of cell”. N = 60.
